# Association of Plasma Aß Peptides with Blood Pressure in the Elderly

**DOI:** 10.1371/journal.pone.0018536

**Published:** 2011-04-15

**Authors:** Jean-Charles Lambert, Jean Dallongeville, Kathryn A. Ellis, Susanna Schraen-Maschke, James Lui, Simon Laws, Julie Dumont, Florence Richard, Dominique Cottel, Claudine Berr, David Ames, Colin L. Masters, Christopher C. Rowe, Cassandra Szoeke, Christophe Tzourio, Jean-François Dartigues, Luc Buée, Ralph Martins, Philippe Amouyel

**Affiliations:** 1 INSERM U744, Lille, France; 2 Institut Pasteur de Lille, Lille, France; 3 Université Lille Nord de France, UDSL, Lille, France; 4 Department of Psychiatry, University of Melbourne, St George's Hospital, Victoria, Australia; 5 Mental Health Research Institute, University of Melbourne, Parkville, Victoria, Australia; 6 National Ageing Research Institute, Parkville, Victoria, Australia; 7 INSERM U837, Lille, France; 8 Centre of Excellence for Alzheimer's Disease Research and Care, Edith Cowan University, Joondalup, Western Australia, Australia; 9 Sir James McCusker Alzheimer's Research Unit, Perth, Western Australia, Australia; 10 Centre Hospitalier Régional Universitaire, Lille, France; 11 INSERM, U888, Université de Montpellier 1, Montpellier, France; 12 Centre for Neurosciences, University of Melbourne, Parkville, Victoria, Australia; 13 Austin Health, Heidelberg, Victoria, Australia; 14 Australian Commonwealth Scientific and Research Organisation (CSIRO), Parkville, Victoria, Australia; 15 INSERM U708, Paris, France; 16 INSERM U593, Victor Segalen University, Bordeaux, France; University of Swansea, United Kingdom

## Abstract

**Background:**

Aß peptides are often considered as catabolic by-products of the amyloid ß protein precursor (APP), with unknown physiological functions. However, several biological properties have been tentatively attributed to these peptides, including a role in vasomotion.

We assess whether plasma Aß peptide levels might be associated with systolic and diastolic blood pressure values (SBP and DBP, respectively).

**Methodology/Principal Findings:**

Plasma Aß_1-40_ and Aß_1-42_ levels were measured using an xMAP-based assay in 1,972 individuals (none of whom were taking antihypertensive drugs) from 3 independent studies: the French population-based 3C and MONA-LISA (Lille) studies (n = 627 and n = 769, respectively) and the Australian, longitudinal AIBL study (n = 576). In the combined sample, the Aß_1-42_/ Aß_1-40_ ratio was significantly and inversely associated with SBP (p = 0.03) and a similar trend was observed for DBP (p = 0.06). Using the median age (69) as a cut-off, the Aß_1-42_/Aß_1-40_ ratio was strongly associated with both SBP and DBP in elderly individuals (p = 0.002 and p = 0.03, respectively). Consistently, a high Aß_1-42_/ Aß_1-40_ ratio was associated with a lower risk of hypertension in both the combined whole sample (odds ratio [OR], 0.71; 95% confidence interval [CI], 0.56-0.90) and (to an even greater extent) in the elderly subjects (OR, 0.53; 95% CI, 0.37–0.75). Lastly, all these associations appeared to be primarily driven by the level of plasma Aß_1-40_.

**Conclusion:**

The plasma Aß_1-42_/Aß_1-40_ ratio is inversely associated with SBP, DBP and the risk of hypertension in elderly subjects, suggesting that Aß peptides affect blood pressure *in vivo*. These results may be particularly relevant in Alzheimer's disease, in which a high Aß_1-42_/Aß_1-40_ plasma ratio is reportedly associated with a decreased risk of incident disease.

## Introduction

Aß peptides are the main component of ß-amyloid deposits in the brains of Alzheimer's disease (AD) patients. Many different cell types from the brain and the peripheral tissues produce these peptides. They are catabolic by-products of the amyloid ß protein precursor (APP) and do not have a known physiological function. However, several lines of evidence suggest that Aß peptides may have biological functions by acting as ligands for various receptors and other molecules [1]–[3]. The peptides are transported between tissues and across the blood brain barrier via complex trafficking pathways [4]. Lastly, at physiological concentrations, the peptides may possess neurotrophic [5], antioxidant [6], platelet aggregation modulation [7], antimicrobial [8] and/or vasoconstriction properties [9].

With respect to vascular tone, Aß peptides are produced by the vascular smooth muscular cells (SMCs) [10] involved in blood pressure (BP) control and are known to have vasoactive properties [11]. Indeed, in *in vitro* studies, Aß peptides enhance constriction of isolated vessels via the release of endothelin 1 [12], a vasoactive peptide which produces smooth muscle contraction *in vivo* [11]. Taken as a whole, these observations suggest that the Aß peptides may affect BP control. Interestingly, the Aß peptides decrease cerebral blood flow and volume in rodents [13]–[15].

In the present study, we hypothesized that plasma Aß peptide concentrations may be associated with variations in systolic and/or diastolic blood pressure values (SBP and DBP, respectively). To this end, we analysed a pooled analysis of 1972 individuals from three independent cohorts in which plasma Aß_1-40_ and Aß_1-42_ concentrations were available.

## Methods

The three samples were selected according to the availability of (i) plasma Aß concentration assays using the same method (the INNO-BIA plasma Aß forms assay; this point is of particular importance, since the assay methodology can significantly influence interpretation of the data [16]), (ii) SBP and DBP measurements; (iii) information on demographic variables, smoking and medication use.

### Populations

Written, informed consent was obtained from study participants. The study protocols for all populations were reviewed and approved by the appropriate independent ethics committees in each country. The institutional ethics committees of Austin Health, St Vincent's Health, Hollywood Private Hospital and Edith Cowan University granted ethics approval for the AIBL study. The institutional ethics committees of the Kremlin-Bicetre Hospital granted ethics approval for the 3C study. The institutional ethics committees of the Lille Hospital granted ethics approval for the MONA-LISA study.

The 3C Study is a population-based, prospective study of the relationship between vascular factors and dementia [17]. It has been carried out in three French cities: Bordeaux (southwest France), Montpellier (southern France) and Dijon (central eastern France). A sample of non-institutionalised, over-65 subjects was randomly selected from the electoral rolls of each city between January 1999 and March 2001. In the present work, the study population was based on a sub-cohort of 1254 subjects randomly selected from the source sample totalling 8,414 individuals (i.e. a sampling ratio of 15%) stratified by centre, 5-year age class and gender. Aß plasma concentrations were measured in the whole sample [18]. Individuals taking antihypertensive drugs were excluded from our analysis (n = 615). Individuals for whom at least one Aß plasma concentration or co-variable measurement was missing were also excluded (n = 4), together with individuals exhibiting at least one aberrant Aß plasma concentration measurement (n = 8). These selection steps allowed us to define a sample of 627 individuals.

The MONA-LISA (LILLE) study is an epidemiological, cross-sectional, population-based study performed in the Lille urban area in northern France. Inhabitants aged 35-74 years were randomly sampled from electoral rolls after stratification by town size, gender and 10-year age groups (n = 1,602) [19]. Only individuals older than 45 years old were selected (n = 1217) and blood samples were obtained from 1201 individuals. Our analysis excluded individuals taking antihypertensive drugs (n = 422), those for whom at least one Aß plasma concentration, SBP, DBP or co-variable measurement was also missing (n = 7) and those exhibiting at least one aberrant Aß plasma concentration measurement (n = 3). These selection steps allowed us to define a sample of 769 individuals.

The Australian Imaging Biomarkers and Lifestyle (AIBL) study of ageing has been described elsewhere [20]. It is a longitudinal study performed in Perth and Melbourne (Australia). A total of 1,112 volunteers constituted the AIBL inception cohort. Our analysis excluded individuals taking antihypertensive drugs (n = 375), those for whom at least one Aß plasma concentration, SBP, DBP or co-variable measurement was also missing (n = 147) and those exhibiting at least one aberrant Aß plasma concentration measurement (n = 14). Again, these selection steps enabled us to define a sample of 576 individuals from the AIBL cohort.

### Amyloid beta peptide assay

Fasting plasma samples were collected in tubes containing sodium EDTA as an anticoagulant. Following centrifugation, plasma samples were aliquoted into polypropylene tubes, stored at –80°C and only thawed immediately prior to Aß quantification. The plasma Aß peptide assay was performed using the INNO-BIA plasma Aß forms assay (Innogenetics, Ghent, Belgium) based on the multiplex xMAP technique with a LABScan-100 system (Luminex BV, The Netherlands). The 3C and MONA-LISA (LILLE) studies were analyzed in the same centre (INSERM U837, Alzheimer & Tauopathies, Lille, France).

### Blood pressure measurements and co-variables

During inclusion in the 3C study, BP was measured twice after 5 minutes in the seated position by using a standard cuff placed around the right arm and an electronic monitor (OMRON M4). In the MONA-LISA (LILLE) population, SBP and DBP were measured after the subject had been seated for at least 10 min with an automatic sphygmomanometer (OMRON 705IT) and an appropriately sized cuff, with the arm at heart level. In the AIBL study, BP for each participant was measured between 8.15 am and 9.30 am and after 10 minutes in the seated position by using the Welch Allyn “DuraShock” handheld unit (DS65). If a measurement was high (>140/90) or low, the procedure was repeated after 10 minutes.

The average of two measurements (available for 84% of the study sample) was used for analysis, whenever possible. Hypertension was defined as a SBP ≥140 mm Hg or DBP ≥90 mmHg (n = 337 in the 3C sample, n = 307 in the MONA-LISA (LILLE) sample and n = 270 in the AIBL sample).

Age, centre and gender were always used as adjusting factors. Several other co-variables were also considered as potential confounders: smoking status (current or not), plasma cholesterol (total, high-density lipoprotein), creatinine levels and body mass index (BMI, as defined by the Quetelet equation).

### Statistics

The data were analysed using SAS statistical software (release 9.1, SAS Institute Inc., Cary NC, USA). In each centre, each quantitative variable was transformed into a z-score (equal to (observed value minus the sample mean), divided by the sample standard deviation). The relationships between the Aß_1-40_, Aß_1-42_ and Aß_1-42_/Aß_1-40_ z-scores on one hand and the SBP or DBP z-scores on the other were assessed using a general linear model (GLM) adjusted for age, centre and gender (model 1). Analyses were subsequently adjusted for other confounders, as defined below: smoking status, total cholesterol z-score, HDL z-score, creatinine z-score and BMI z-score (model 2). Interactions between Aß_1-42_/Aß_1-40_ z-scores, SBP or DBP z-scores and age or gender were tested in a GLM adjusted as for model 2.

We analysed the association of Aß_1-40_, Aß_1-42_ and Aß_1-42_/Aß_1-40_ z-scores with the risk of hypertension. Aß_1-40_, Aß_1-42_ and Aß_1-42_/Aß_1-40_ z-scores tertiles were defined and the lowest was used as a reference in a logistic regression model. Odds ratios were systematically adjusted for centre, age, gender, smoking status, cholesterol (total, high-density lipoprotein), creatinine levels and BMI z-scores (model 2).

## Results

The characteristics of the three independent, constituent samples are presented in Table 1. There was a statistically significant, inverse association between plasma Aß_1-42_/Aß_1-40_ and SBP (p = 0.03; Table 2). A similar trend was observed for DBP (p = 0.06; Table 2). We also looked at whether or not Aß_1-42_/Aß_1-40_ was associated with the risk of hypertension. In fact, individuals in the upper Aß_1-42_/Aß_1-40_ tertile had a 1.4-fold lower risk of hypertension than subjects in the lower tertile (Table 3).

**Table 1:** Baseline sociodemographic variables and potential confounding factors in the 3C, ABLI and MONA-LISA (LILLE) populations (individuals not taking antihypertensive drugs; for details, see the Materials and Methods section).

	3C study (n = 627)	AIBL study (n = 576)	MONA-LISA study (n = 769)
Age (years)	73.1±5.3	71.6±7.8	58.1±8.2
% women	59.7%	57.8%	49.4%
Smoking (% current)	6.7%	3.0%	18.6%
Body mass index (kg/m^2^)	24.9±3.5	25.5±4.1	26.5±4.5
Plasma HDL cholesterol (mmol/L)	1.67±0.41	1.70±0.44	1.50±0.39
Plasma cholesterol (mmol/L)	6.0±1.0	5.7±1.1	5.87±1.1
Plasma creatinine	80.5±15.6	81.1±17.3	85.0±15.8
SBP (mmHg)	141.9±20.4	136.5±14.7	136.5±18.6
DBP (mmHg)	81.3±11.0	78.0±9.3	82.2±10.6
Plasma Aß_1-40_ (pg/ml)	227.8±48.0	153.6±40.4	205.4±42.8
Plasma Aß_1-42_ (pg/ml)	37.5±10.3	31.3±10.0	36.7±11.45
Plasma Aß_1-42_/ Aß_1-40_	0.169±0.049	0.209±0.059	0.184±0.069

**Table 2:** Associations between plasma Aß peptides and SBP & DBP values.

Combined sample	Model 1		Model 2	
**Aß_1-40_**	ß	p	ß	p
SBP z-score	+0.006±0.023	0.80	+0.006±0.023	0.80
DBP z-score	+0.011±0.026	0.65	+0.005±0.023	0.83
**Aß_1-42_**	ß	p	ß	p
SBP z-score	−0.036±0.021	0.09	−0.039±0.021	0.10
DBP z-score	−0.023±0.022	0.31	−0.031±0.022	0.16
**Aß_1-42_/Aß_1-40_**	ß	p	ß	p
SBP z-score	−0.044±0.022	0.04	−0.045±0.021	0.03
DBP z-score	−0.037±0.022	0.10	−0.040±0.022	0.06

Data are ß coefficients ± 95% CI.

Model 1: Adjusted for age, gender, centre.

Model 2: Adjusted for age, gender, centre, smoking status, total cholesterol z-score, HDL z-score, creatinine z-score and BMI z-score.

**Table 3:** Associations between the plasma Aß_ 1-42_/A ß_1-40_ ratio and hypertension.

	A ß_ 1-42_/A ß_ 1-40_ z-score			
Risk of hypertension	1^st^ tertile	2^nd^ tertile	3^rd^ tertile	p
Whole sample	1.00 (ref)	0.81 (0.65–1.03)	0.71 (0.56–0.90)	0.004
>69 years of age	1.00 (ref)	0.69 (0.49–0.98)	0.53 (0.37–0.75)	0.0003

The odds ratio (95% CI) for hypertension (n = 914) was adjusted for age, gender, centre, smoking status, total cholesterol z-score, HDL z-score, creatinine z-score and BMI z-score.

We next searched for potential interactions between age, gender and the associations described above. We observed significant interactions with age when analysing the association between SBP or the risk of hypertension and plasma Aß_1-42_/Aß_1-40_ (p = 0.02 and p = 0.04, respectively). These observations are particular relevant, since BP and plasma Aß concentrations are already known to be strongly age-dependent [18], [21].

We thus stratified the combined sample using the median age (>69) as a cut-off and observed more pronounced inverse associations between BP levels and Aß_1-42_/Aß_1-40_ in the elderly individuals (Table 4). Importantly, the association of Aß_1-42_/Aß_1-40_ with BP levels appeared to be primarily linked to plasma Aß_1-40_ levels (Table 4). Similarly, this association was even more pronounced in the elderly, where individuals in the upper Aß_1-42_/Aß_1-40_ tertile had a 2-fold lower risk of hypertension (Table 3). The plasma Aß_1-42_/Aß_1-40_ ratio's associations with hypertension again appeared to be mainly driven by plasma Aß_1-40_ in the oldest subjects. Individuals in the upper Aß_1-40_ z-score tertile had a greater risk of suffering from hypertension (OR = 1.61, 95% CI [1.09–2.39], p = 0.01), whereas no association with the Aß_1-42_ z-score was found (OR = 1.03, 95% CI [0.74–1.45], p = 0.85).

**Table 4:** Associations between plasma Aß peptides and SBP & DBP values.

≤69 years of age	Model 1		Model 2	
**Aß_1-40_**	ß	p	ß	p
SBP z-score	−0.046±0.030	0.12	−0.049±0.029	0.10
DBP z-score	−0.025±0.031	0.41	−0.035±0.030	0.24
**Aß_1-42_**	ß	p	ß	p
SBP z-score	−0.028±0.030	0.34	−0.036±0.030	0.23
DBP z-score	−0.008±0.031	0.80	−0.032±0.031	0.54
**Aß_1-42_/Aß_1-40_**	ß	p	ß	p
SBP z-score	+0.013±0.022	0.56	+0.006±0.022	0.78
DBP z-score	−0.010±0.024	0.67	−0.013±0.022	0.57

>69 years of age				
**Aß_1-40_**	ß	p	ß	p
SBP z-score	+0.099±0.034	0.004	+0.098±0.035	0.005
DBP z-score	+0.066±0.035	0.06	+0.066±0.035	0.07
**Aß_1-42_**	ß	p	ß	p
SBP z-score	−0.032±0.031	0.30	−0.039±0.031	0.21
DBP z-score	−0.028±0.031	0.38	−0.032±0.031	0.30
**Aß_1-42_/Aß_1-40_**	ß	p	ß	p
SBP z-score	−0.090±0.030	0.003	−0.092±0.030	0.002
DBP z-score	−0.064±0.031	0.04	−0.067±0.031	0.03

Data are ß coefficients ± 95% CI.

Model 1: Adjusted for age, gender, centre.

Model 2: Adjusted for age, gender, centre, smoking status, total cholesterol z-score, HDL z-score, creatinine z-score and BMI z-score.

Importantly, all the reported associations appeared homogenous and in the same direction in the three samples when analysed separately (Table S1 and S2) and remained significant when pairs of populations were compared in a sensitivity analysis (data not shown).

## Discussion

Here, we have shown that an elevated plasma Aß_1-42_/Aß_1-40_ ratio is significantly associated with low SBP and DBP values. In the elderly, we estimate that a 0.01 unit increment in Aß_1-42_/Aß_1-40_ was associated with a 0.29±0.09 mmHg decline in SBP and a 0.10±0.05 mmHg decline in DBP. Consistently, an elevated Aß_1-42_/Aß_1-40_ plasma ratio was also associated with a lower risk of hypertension in the elderly.

Importantly, the plasma Aß_1-42_/Aß_1-40_ ratio's associations with SBP, DBP and hypertension appeared to be mainly driven by plasma Aß_1-40_ in the oldest subjects. Our observation of an association between plasma Aß_1-40_ and SBP agrees with the report of a similar trend (in a small sample) by Abdullah et al [22]. The mechanisms underlying this preferential association may be related to the Aß_1-40_ peptide's properties on vascular vessels. Earlier studies have shown that Aß_1-40_ peptides can constrict cerebral blood vessels *in vitro* ^9^ and decrease cerebral flow and cerebral blood volume *in vivo* [10]–[12]. and that Aß_1-40_ has greater vasoconstriction effects on the cerebral vasculature than Aß_1-42_ does [12]. Furthermore, in rodents, injection of Aß_1-40_ into the tail modulates cerebral blood flow and volume, suggesting that Aß peptides have a direct impact on blood pressure. Finally, Aß peptides have been described to potentially modulate the vasoactivity of the rat aorta [23]. Thus, by extension our observation of an association between an elevated Aß_1-42_/Aß_1-40_ ratio and low SBP may be related to the properties of Aß_1-40_ on vascular wall in the elderly. *In vitro* and *in vivo* experiments will be needed to underpin this epidemiological observation and to extend knowledge of the Aß peptides' vasoactivity from the cerebral vasculature to the vascular system as a whole. Alternatively, we cannot rule out the possibility that the plasma Aß_1-42_/Aß_1-40_ ratio is merely a marker of other parameters involved in BP variations or that the plasma Aß_1-42_/Aß_1-40_ association with BP is a consequence of BP variations by themselves. These possibilities may help explain the stronger association of the plasma Aß_1-42_/Aß_1-40_ ratio with SBP and DBP in the elderly individuals. Age-related arterial wall stiffening may lead indirectly to subtle changes in APP metabolism in the SMCs (one of the main non-brain cell types able to produce Aß peptides). Again, only *in vitro* and *in vivo* experiments will be able to clarify this question.

Nonetheless, and notwithstanding the consistent effects that we have observed, our study suffered from a number of limitations. Firstly, quantification of Aß peptides in plasma is not fully standardized and it varied from one centre to another (Table 1). Even though centre-to-centre variations are well known for quantitative variables, we cannot rule out the possible presence of assay-related of bias. Therefore, in order to minimize between-centre variability, we transformed the data in to z-scores prior to our statistical analyses. Secondly, it is still unclear whether the assay used here does indeed quantify all the various free, bound, monomeric and oligomeric forms of plasma Aß_1-40_ and Aß_1-42_ peptides. Accordingly, we may only have a partial picture of the Aß_1-40_ and Aß_1-42_ concentrations in plasma - a picture which is also likely to be influenced by sample conditioning, storage and analyses. In order to minimize this problem, we analysed three independent cohorts in which the same plasma Aß peptide assay method had been used. Interestingly, the assay performed in the present study uses xMAP technology to quantify several epitopes and thus several different Aß species. Furthermore, we observed a strong correlation between plasma Aß_1-40_ and Aß_1-42_ in all the populations analysed (data not shown) - indicating that the plasma Aß peptide concentrations are representative of the physiological processes leading to Aß peptide production (i.e. APP metabolism). Furthermore, sensitivity analyses indicated that the observed results are homogeneous for the elderly individuals in the different studies and support the existence of a real impact of Aß peptides on BP values and hypertension (Tables S1 and S2).

Our data may be of particular interest in the field of dementia. On the epidemiological level, an increased risk of dementia in individuals with high BP (and especially very high SBP) has been reported [24], although there is no clear consensus to indicate that raised BP in later life is a risk factor in dementia [25]–[27]. Furthermore, use of antihypertensive agents was suggested to reduce the risk of dementia and cognitive decline observed in clinical trials [28]. Interestingly, we and others have observed that an elevated Aß_1-42_/Aß_1-40_ ratio is strongly associated with a decreased risk of incident Alzheimer's disease and mixed/vascular dementia [18], [29]. Consequently, we can justifiably postulate that high plasma Aß_1-42_/Aß_1-40_ may reduce and/or delay the risk of developing dementia in the elderly by decreasing SBP and lowering the risk of hypertension. Our data might be also consistent with the finding that plasma Aß_1-40_ is associated with microvascular brain injury in subjects with AD, mild cognitive impairment or cerebral amyloid angiopathy [30].

In conclusion, our data support the potential vasoactive properties of the Aß peptides and suggest that the latter are able to subtly modulate BP in the elderly. Furthermore, these observations may offer new opportunities for better understanding the vascular component of dementia in general and Alzheimer's disease in particular.

## Supporting Information

Table S1 Associations between plasma Aß peptides and SBP & DBP values in the elderly participants in the 3C (n = 445), MONA-LISA (LILLE) (n = 102) and AIBL (n = 323) studies. Adjusted for age, gender, centre, smoking status, total cholesterol z-score, HDL z-score, creatinine z-score and BMI z-score.
(DOC)

Table S2 Associations between plasma Aß_1-42_/Aß_1-40_ ratio and hypertension in the elderly. Odds ratio (95% CI) for hypertension in the 3C study (n = 265), in the MONA-LISA (LILLE) study (n = 58) and the AIBL study (n = 180). Adjusted for age, gender, centre (when necessary), smoking status, total cholesterol z-score, HDL z-score, creatinine z-score and BMI z-score.
(DOC)
