# Supplementary material for: Association of Plasma Aß Peptides with Blood Pressure in the Elderly
Source: PLoS One. 2011 Apr 15;6(4):e18536. doi: 10.1371/journal.pone.0018536 (PMC3078119; doi:10.1371/journal.pone.0018536)
Supplement: Table S1 — Associations between plasma Aß peptides and SBP & DBP values in the elderly participants in the 3C (n = 445), MONA-LISA (LILLE) (n = 102) and AIBL (n = 323) studies. Adjusted for age, gender, centre, smoking status, total cholesterol z-score, HDL z-score, creatinine z-score and BMI z-score. (DOC) [file pone.0018536.s001.doc]

**Table S1**

| Elderly population | SBP | | DBP | |
| --- | --- | --- | --- | --- |
|  |  |  |  |  |
| **Aß _1-40_** | ß | p | ß | p |
| 3C | +0.070 ± 0.047 | 0.14 | +0.077 ± 0.049 | 0.12 |
| MONA-LISA (LILLE) | +0.148 ± 0.110 | 0.17 | +0.016 ± 0.101 | 0.87 |
| AIBL | +0.146 ± 0.061 | 0.02 | +0.086 ± 0.061 | 0.16 |
|  |  |  |  |  |
| **Aß_1-42_** | ß | p | ß | p |
| 3C | -0.050 ± 0.046 | 0.28 | -0.017 ± 0.047 | 0.73 |
| MONA-LISA (LILLE) | -0.180 ± 0.074 | 0.02 | -0.091 ± 0.070 | 0.20 |
| AIBL | +0.064 ± 0.052 | 0.22 | - 0.012 ± 0.053 | 0.81 |
|  |  |  |  |  |
| **Aß_1-42_/Aß_1-40_** | ß | p | ß | p |
| 3C | -0.104 ± 0.050 | 0.04 | -0.085 ± 0.050 | 0.10 |
| MONA-LISA (LILLE) | -0.152 ± 0.060 | 0.01 | -0.046 ± 0.057 | 0.42 |
| AIBL | -0.041 ± 0.051 | 0.42 | -0.076 ± 0.052 | 0.15 |
