# Supplementary material for: Association of Plasma Aß Peptides with Blood Pressure in the Elderly
Source: PLoS One. 2011 Apr 15;6(4):e18536. doi: 10.1371/journal.pone.0018536 (PMC3078119; doi:10.1371/journal.pone.0018536)
Supplement: Table S2 — Associations between plasma Aß1-42/Aß1-40 ratio and hypertension in the elderly. Odds ratio (95% CI) for hypertension in the 3C study (n = 265), in the MONA-LISA (LILLE) study (n = 58) and the AIBL study (n = 180). Adjusted for age, gender, centre (when necessary), smoking status, total cholesterol z-score, HDL z-score, creatinine z-score and BMI z-score. (DOC) [file pone.0018536.s002.doc]

**Table S2**

|  |  |  |  |  |
| --- | --- | --- | --- | --- |
|  | Aß_1-42_/Aß_1-40_ z-score | | |  |
| Risk of hypertension | 1^st^ tertile | 2^nd^ tertile | 3^rd^ tertile | p |
| 3C | 1.00 (ref) | 0.74 (0.45-1.22) | 0.53 (0.33-0.86) | 0.007 |
| MONA-LISA (LILLE) | 1.00 (ref) | 0.32 (0.10-1.00) | 0.16 (0.04-0.53) | 0.004 |
| AIBL | 1.00 (ref) | 0.76 (0.42-1.38) | 0.63 (0.36-1.12) | 0.17 |
|  |  |  |  |  |
